# Phenolic Compounds in Honey and Their Associated Health Benefits: A Review

**DOI:** 10.3390/molecules23092322

**Published:** 2018-09-11

**Authors:** Danila Cianciosi, Tamara Yuliett Forbes-Hernández, Sadia Afrin, Massimiliano Gasparrini, Patricia Reboredo-Rodriguez, Piera Pia Manna, Jiaojiao Zhang, Leire Bravo Lamas, Susana Martínez Flórez, Pablo Agudo Toyos, José Luis Quiles, Francesca Giampieri, Maurizio Battino

**Affiliations:** 1 Dipartimento di Scienze Cliniche Specialistiche ed Odontostomatologiche (DISCO)-Sez. Biochimica, Facoltà di Medicina, Università Politecnica delle Marche, 60131 Ancona, Italy; danila.cianciosi@gmail.com (D.C.); tamara.forbe@gmail.com (T.Y.F.-H.); dolla.bihs@gmail.com (S.A.); m.gasparrini@univpm.it (M.G.); preboredo@uvigo.es (P.R.-R.); p.piera@hotmail.it (P.P.M.); zh.jojo@yahoo.com (J.Z.); 2 Departamento de Química Analítica y Alimentaria, Grupo de Nutrición y Bromatología, Universidade de Vigo, 32004 Ourense, Spain; 3 Center for Nutrition & Health, Universidad Europea del Atlántico (UEA), 39011 Santander, Spain; leire.bravo@uneatlantico.es (L.B.L.); susana.martinez@uneatlantico.es (S.M.F.); pablo.agudo@uneatlantico.es (P.A.T.); 4 Department of Physiology, Institute of Nutrition and Food Technology “Jose Mataix”, Biomedical Research Centre, University of Granada, Armilla, 18100 Granada, Spain; jlquiles@ugr.es

**Keywords:** honey, antioxidants, polyphenols, antimicrobial activities, cancer, diabetes, disease prevention

## Abstract

Honey is a natural substance appreciated for its therapeutic abilities since ancient times. Its content in flavonoids and phenolic acids plays a key role on human health, thanks to the high antioxidant and anti-inflammatory properties that they exert. Honey possesses antimicrobial capacity and anticancer activity against different types of tumors, acting on different molecular pathways that are involved on cellular proliferation. In addition, an antidiabetic activity has also been highlighted, with the reduction of glucose, fructosamine, and glycosylated hemoglobin serum concentration. Honey exerts also a protective effect in the cardiovascular system, where it mainly prevents the oxidation of low-density lipoproteins, in the nervous system, in the respiratory system against asthma and bacterial infections, and in the gastrointestinal system. A beneficial effect of honey can also be demonstrated in athletes. The purpose of this review is to summarize and update the current information regarding the role of honey in health and diseases.

## 1. Introduction

Oxidative stress is the basis of structural and functional damage to the main biomolecules such as nucleic acids, lipids, and proteins. In fact, these injuries lead to the development of many diseases, such as cancer, metabolic disorders, and cardiovascular dysfunctions. The imbalance created between the production of free radicals and antioxidant defense can occur not only in pathological situations, but also in some physiological conditions such as intense physical activity [1]. Exogenous intake of antioxidant compounds through the diet can counteract the effect of oxidant molecules such as free radicals, reducing oxidant stress [2]. Honey is a natural substance that bees produce from honeydew or nectar of flowers. Bees collect nectar or honeydew, and transport them into the hive, which begins the processes that lead to their transformation into honey: the concentration and the enzymatic conversion of sugar. Honey composition is closely related to its botanical origin, and to the processing and environmental conditions [3].

**Figure 1:**
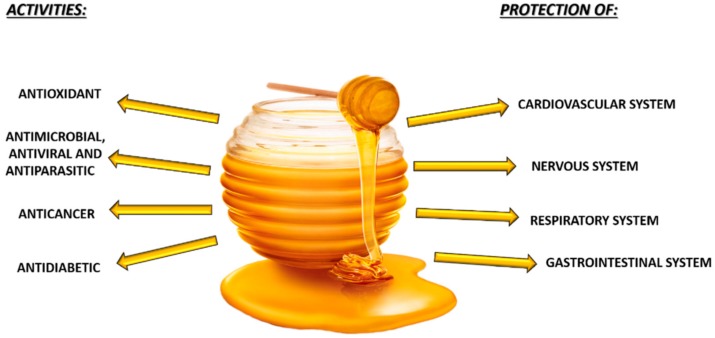
Beneficial effect of honey consumption.

Since ancient times, honey has been not only considered a food or a sweetener, but it was also used as a medicine for stimulating healing of wound, tissue regeneration, and alleviating gastrointestinal disorders, gingivitis, and various other pathologies. The therapeutic effect of honey results from the presence of various antioxidant molecules, including phenolic compounds, such as flavonoids and phenolic acids [4]. Several in vitro and in vivo studies have demonstrated the antimicrobial, antiviral, antifungal, anticancer, and antidiabetic activity of honey. In addition, the protective effect on cardiovascular, nervous, respiratory, and gastrointestinal systems has been also proven [5,6]. A protective effect of honey was also observed in physiological condition characterized by high levels of free radicals, such as those of athletes practicing different sports [7] (Figure 1).

The effect of honey on human health depends on the bioavailability of the phytochemical compounds, and on their methods of absorption and metabolization. The aim of the present review is to summarize and update recent evidence, obtained from in vitro and in vivo studies, on the potential of honey in maintaining human well-being, and preventing the most common diseases.

## 2. Chemical and Phytochemical Composition

Honey contains about 180 types of different compounds, including water, sugars, free amino acids, proteins, enzymes, essential minerals, vitamins, and various phytochemicals (Table 1) [8]. The composition, taste and color of the different honey depend on the type of flower source, the geographical area, the climate, and the different species of bees involved in honey production, which is also conditioned by the processing techniques and storage [9].

### 2.1. Nutrients

Honey is an important source of macro- and micronutrients. Carbohydrates, mono- and disaccharides, comprise 95% of its dry weight: glucose and fructose are present in larger quantities, and they contribute principally to the energetic value and physical characteristics of honey, such as hygroscopicity, granulation, and viscosity. The concentration of glucose and fructose and the relationship between them is one of the main classification parameters in monofloral honeys. Among carbohydrates, maltose, sucrose, turanose, isomaltose, cellobiose, isopanose, and many others in different quantities can be also found [10].

The disaccharides and trisaccharides present in honey are hydrolyzed into monosaccharides from different types of enzymes, such as invertases and α-glucosidases [11]. These sugars are subjected to chemical changes during honey storage: a long or wrong conservation can lead to the formation of undesirable compounds derived from pentoses (furfural) and hexoses (5-hydroxymethylfurfural (5-HMF)). Usually these products derive from the Maillard reaction, and they are used to test the quality of honey, indicating a possible exposure to high temperatures or a prolonged storage time [12].

Minor amounts of proteins are present, mainly in the form of enzymes and free amino acids, except for asparagine and glutamine. Proline is the main amino acid in honey (50–85%), resulting primarily from the salivary secretions of honeybees (*Apis mellifera* L.); it is used as a parameter to evaluate the degree of honey maturation. Other amino acids are alanine, phenylalanine, tyrosine, glutamic acid, isoleucine, and leucine [13]. 

In honey, there is also a variable amount of essential minerals (about 0.2% of its dry weight), which varies according to its botanical origin, environmental conditions and processing. Among the most represented minerals, potassium, calcium, copper, iron, magnesium, manganese, phosphorus, sodium, zinc and selenium can be found. Honey also contains small amounts of vitamins, such as ascorbic acid (C), thiamine (B1), riboflavin (B2), niacin (B3), pantothenic acid (B5), and pyridoxine (B6) [5]. All the vitamins of the complex B derive mainly from pollen, and together with vitamin C, it can be influenced by commercial and industrial processes, such as filtration and by oxidation reactions carried out by glucose oxidase [14].

### 2.2. Enzymes and Organic Acids

A portion of proteins present in the honey consists essentially of enzymes derived from pollen, nectar, and bees. The main enzymes are diastase, glucose oxidase, and invertase. Diastases are amylolytic enzymes, such as α-amylases that hydrolyse the chains of starch producing dextrin and β-amylases that lead to maltose formation, and whose activity is an important factor for honey’s quality. Glucose oxidase converts glucose into δ-gluconolactone, which is hydrolyzed to gluconic acid, the principal acid in honey and hydrogen peroxide (H_2_O_2_), responsible of the antimicrobial activity of honey. The invertase has the ability to hydrolyse sucrose in glucose and fructose [15].

All types of honey have some acidity, because of the presence, although low, of organic acids; these acids contribute both to the honey flavor and to its antimicrobial activity, as well as to the stability of this food matrix. The most important is gluconic acid, followed by aspartic acid, citric, acetic, formic, fumaric, galacturonic, malonic, formic, acetoglutaric, gluconic, glutamic, butyric, glutaric, butyric, shikimic, propionic, pyruvic glyoxylic, 2-hydroxybutyric, α-hydroxyglutaric, isocitric, lactic, malic, methylmalonic, quinic, succinic, tartaric, oxalic and others [16].

### 2.3. Phenolic Compounds

**Figure 2:**
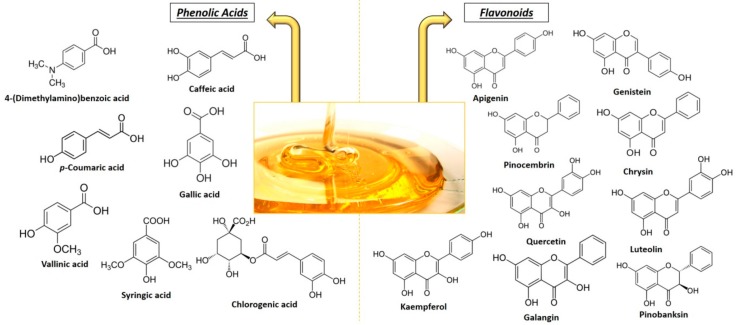
Most common phenolic compounds identified in honey.

Polyphenols are a heterogeneous class of chemical compounds that can be divided into flavonoids (flavonols, flavones, flavanols, flavanones, anthocyanidin, chalcones, and isoflavones) and non-flavonoids (phenolic acids). All these compounds are often the product of secondary plant metabolism and are characterized by the presence of multiple phenolic groups that are associated with more or less complex structures. The secondary metabolites differ from the primary (chlorophyll, amino acids, and simple carbohydrates) because, although they have important ecological functions, they do not mediate in the processes of assimilation, respiration, transport, and differentiation of the plants. The phenolic composition in honey mainly depends on its floral origin; in fact, it can be used as a tool for classification and authentication, especially in the case of unifloral varieties. The most common phenolic compounds in honey are shown in Figure 2, and in Table 2, it is possible to compare the phenolic compounds identified in the different types of honey that are mentioned in this review [17,18,19,20,21,22,23,24,25,26,27,28].

These substances have been recognized as the main responsible for the antioxidant activity of honey that is mainly associated with the ability of free radical scavengers, through the formation of more stable and less toxic molecules. Phenolic compounds stabilize free radicals when they give off hydrogen from one of their hydroxyl group; the degree of activity is related to the number of their hydroxyl groups [29].

**Figure 3:**
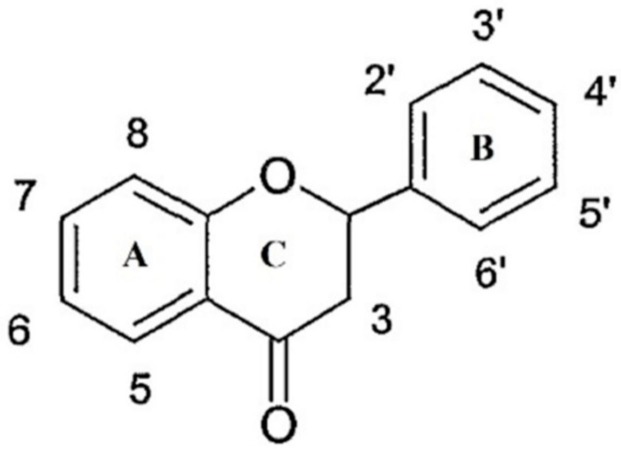
Basic flavonoid structure.

The flavonoids are natural chemical compounds with low molecular weight, and they are mainly water-soluble. They are formed by two benzene rings, alternated by a linear chain of three atoms of carbon (C6-C3-C6); this structure often rearranges itself to form three rings with 15 carbon atoms called A, B and C (Figure 3). Generally these compounds have at least two phenolic groups (OH), and are often associated with sugars (glycosides), mainly glucose together with xylose, galactose, rhamnose, arabinose, rutinoside and glucorhamnose; when flavonoids are not associated with sugars they are called aglycones. The flavonoids are then classified according to the degree of oxidation of the C ring in: flavanols, flavones, flavanonols, flavonols, flavanones, isoflavones, anthocyanins, and anthocyanidins. The most abundant ones in honey are flavones, flavanols, and flavonols [30].

Phenolic acids (phenol carboxylic acid) contain a phenolic ring and at least one organic carboxylic acid function; they can be divided according to their structure: C6-C3 (e.g., p-coumaric, ferulic and caffeic acid), C6-C2 (e.g., acetophenones and phenylacetic acids) and C6-C1 structure (e.g., syringic, vanillic and gallic acid). Usually, most of these compounds are bound to the structural components of the plant (cellulose, lignin), but also to other types of organic molecules such as glucose, other sugars, or flavonoids [31].

## 3. Metabolism and Bioavailability of Honey Polyphenols

As mentioned previously, the beneficial effect of honey on human health derives mainly from its content in phenolic compounds. To understand the effects that these phenolic compounds have, it is appropriate to evaluate their absorption and metabolism. There are several factors that can influence the bioavailability of dietary polyphenols, such as environmental factors, food processing, type of matrix, interaction with other compounds (proteins or other polyphenols), the chemical structure of the phenolic compounds, and intestinal factors (composition of microflora). There is no explanation of the process of absorption, metabolism and excretion that is valid for all phenolic compounds [32]. There are studies that have been performed both in vitro and in vivo to try to understand the mechanisms behind the bioavailability of these compounds [33]; only a few studies have specifically investigated the polyphenols derived from honey. One of these studies examined the total amount of polyphenols after honey ingestion in plasma, and it found a minimal quantity compared to that present in honey, demonstrating a very low bioavailability and absorption [34]. 

Most of the studies on phenol metabolism are on flavonoids. A first phase of flavonoid metabolism concerns the hydrolysis reaction that can be performed both by bacterial enzymes present in the intestine, and by two kinds of enzymes present in the small intestine. The β-hydrolysis of the sugar in the glycosylated flavonoids can be in fact catalyzed by two β-endoglucosidases: the lactase phlorizin hydrolase (LPH) and the cytosolic β-glucosidase (CBG) [35]. When LPH, which is found in the brush border of the enterocytes, catalyzes the hydrolytic reaction, the released aglycone can enter more easily to the epithelial cells, due to increased lipophilicity. When the hydrolysis reaction is catalyzed by CBG, the polar glucosides are transported inside the epithelial cells through a sodium-dependent glucose transporter 1 (SGLT1), where they are hydrolyzed [36]. Some studies have shown that some flavonoids can inhibit Na-dependent facilitated diffusion of monosaccharides into intestinal epithelial cells [37]. 

Regarding honey and its flavonoids, they must be taken into account that in this matrix, there are some glucosidases deriving from the salivary glands of bees which could be a further hydrolysis pathway of these compounds, and an explanation to the fact that many flavonoids are found in honey as aglycones. Phenolic aglycones are more easily absorbed by intestinal barriers, increasing their bioavailability, if compared with the same flavonoids glycosylated present in different food matrix [38]. Once absorbed by the intestinal epithelium and before arriving inside the bloodstream, the flavonoids enter the second phase of the metabolism, which leads to the formation of different conjugated products: in particular, sulfotransferases (SULTs) generate sulfates, uridine-5′-diphosphate glucuronosyltransferases (UGTs) allows the formation of glucoronides, the catechol-*O*-methyltransferases (COMTs) produce methylated derivatives. In addition to these metabolic biotransformations that affect the absorption, the bioavailability and the distribution of flavonoids at the cellular and tissue levels are also mediated by some proteins that are associated with multi-resistance (MRP1, MRP2), which are part of the third phase of the metabolism of flavonoids [39]. 

MRP2 is located in the apical membrane of the epithelial cells of the small intestine, and it transports flavonoids back into the intestinal lumen. MRP1 is located in the vascular pole of the enterocytes, and promotes the transport of flavonoids inside the blood cells [40]. Also, MRP3 and the glucose transporter (GLUT2) aid in the transport of these compounds in the portal venous system; once they enter the latter, the metabolites quickly reach the hepatocytes, where the aglycones are transferred in the peroxisomes and in the Golgi apparatus, where they are subjected to further metabolic processes [41].

Some flavonoids contain sugars that are resistant to the action of LPH and CBG, so that they are not absorbed by the small intestine, and they pass directly into the colon where they can be deglycosylated by enterobacteria present in this district. The microflora of the colon also transforms the aglycones into various metabolites that can either be excreted from the feces, or be absorbed by the liver within the enteropathic recirculation of the bile excretion and be further conjugated by specific enzymes as previously explained [42]. Other metabolites, however, after the metabolic changes that occur in the hepatocytes are secreted by some organic acid transporters in the systemic circulation, and they are either absorbed by cells or tissues or expelled by the kidneys [43].

## 4. Oxidative Stress, Antioxidant Activity, and Inflammation

Oxidative stress can be defined as an imbalance between oxidants and antioxidants in favor of oxidants. Oxidative stress produces oxidative damage that can affect various physiological functions. Free radicals and reactive oxygen species (ROS) are the major oxidant agents in cellular systems, and they are involved in aging and in the onset of many kind of diseases [44]. 

ROS and free radicals are physiologically produced in different cellular biochemical reactions that occur in the body, such as in mitochondria for the aerobic production of oxygen [45], in the metabolism of fatty acids [46], in the metabolization of drugs [47], and during the activity of the immune system [48]. On the other hands, free radicals can also be produced by exogenous factors such as pollution, incorrect life habits, UV rays, ionizing radiation, and psychophysical stress from intense physical activity [49]. Antioxidants are molecules that are able to donate an electron to free radicals, neutralizing, decreasing, or eliminating their ability to damage cells and major biomolecules such as nucleic acids, proteins, and lipids [50].

Antioxidants can be endogenous (superoxide dismutase (SOD), alpha lipoic acid (ALA), catalase, coenzyme Q10 (CoQ10), glutathione peroxidase (GPX)) or exogenous, which are taken through the diet. The antioxidant capacity (AOC) is considered to be an indicator of the presence of bioactive compounds in honey. The AOC of honey is given primarily by phenolic compounds, but enzymes, amino acids, and carotenoids also contribute to this ability. Radical scavenging and protection against the lipid peroxidation of honey can reduce and prevent diseases and physiological situations where oxidative stress plays an important role [51]. 

In recent years, it has emerged that oxidative stress plays a fundamental role in the development and propagation of a state of inflammation, leading to the onset of various diseases [52]. Both processes have often been found simultaneously in subjects with diabetes, cancer, cardiovascular, neurodegenerative disorders, and many other disease situations [53].

## 5. Honey and Health and Diseases

### 5.1. Antimicrobial, Antiviral, and Antifungal Activity

The use of honey as antimicrobial is known since ancient times. There are several studies regarding the antibacterial activity of honey (Table 3) [54], which seems to act on both Gram-positive and Gram-negative, although the first are more sensitive. All the studies, summarized in the Table 3 were done using the agar disk-diffusion test and evaluating the minimum inhibitory concentration (MIC), and the minimum bactericidal concentration (MBC) of different types of honey with diverse bacterial agents. In general, monofloral honey has a greater antibacterial effect than multifloral honey [55]. This ability is mainly due to some physical properties of this matrix, such as low water activity (A_w_), high osmotic pressure, low pH, and low protein content, which prevent bacterial growth. In addition to these physical properties, the antimicrobial activity of honey is also due to the glucose oxidase, H_2_O_2_, and to some phenolic compounds such as pinocembrin, syringic acid, and some others compounds [56]. Recent studies are focusing on the presence and role of metylglyoxal (CH3-CO-CH=O), especially in Manuka honey (*Leptospermum scoparium*), since it is believed to be the most responsible for the non-peroxide antibacterial activity of honey [57].

An activity closely linked to antibacterial capacity is wound healing. The ability of honey to sterilize the wounds, stimulate tissue re-growth, and to reduce edema and scar formation, affects simple wounds, burns, diabetic foot ulcers, and pressure ulcers [6,58,59].

Some studies have also shown a certain antiviral activity of honey. It was evaluated that the in vitro effect of Manuka and Clover honey in human malignant melanoma cells (MeWo)-infected with varicella Zoster virus (VZV) isolated from a Zoster vesicle. The results showed a reduction of the viral plaques after the treatment of the cells with both types of honey [60]. A similar effect has also been proven in Madin-Darby canine kidney (MDCK) cells infected with influenza virus (H1N1), treated with different types of honey (Manuka, renge, and Acacia honey). The plaque inhibition assay has been carried out showing a higher antiviral activity of Manuka honey, compared to the other types of honey, and the synergistic effects with some antiviral drugs [61].

It has also been demonstrated an antifungal activity of honey towards different kinds of *Candida* infections (*C. albicans*, *C. glabrata*, and *C. dubliniensis*), and on *Rhodotorula* sp., evaluating the MIC and using the agar disk-diffusion test [62,63].

Finally, the high antimicrobial capacity of honey is also closely linked to the improvement of the gut microbial balance, thanks to the high content of oligosaccharides, which act as a substrate for the growth of prebiotic microorganisms. One study has reported the potential of honey to *Lactobacilli* and *Bifidobacteria*: the vitality and the growth rate of these essential microorganisms in the balance of the gut microbiota increased with the addition of different types of honey [64].

### 5.2. Anticancer Activity

The potential effects of honey on cancer have been investigated both in terms of prevention, and progression and treatment. Most of the studies are in vitro, and they have been carried out on different types of cell lines and different types of honey. Some studies have also been carried out in vivo on mice/rats, inducing or transplanting the tumor [65].

**Figure 4:**
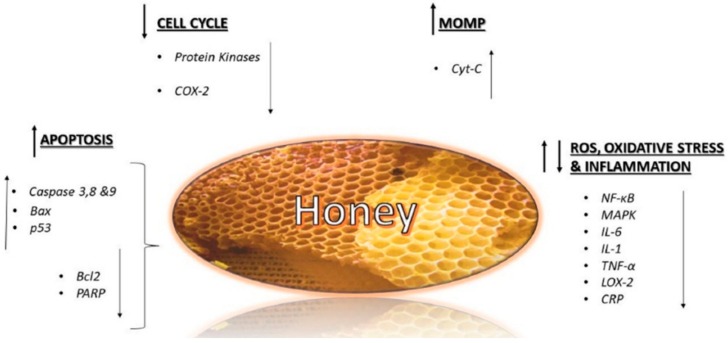
Molecular mechanism involved in anticancer effect of honey.

Honey acts at different stages of cancer, on the initiation, proliferation, and progression. Its antitumoral effects are generally attributed to different mechanisms, such as the induction of apoptosis, cell cycle arrest, the modulation of oxidative stress, the amelioration of inflammation, the induction of mitochondrial outer membrane permeabilization (MOMP), and the inhibition of angiogenesis [66] (Figure 4).

Apoptosis is a programmed cell death process that eliminates damaged cells. Through the up-regulation of some proapoptotic proteins, such as caspase 3, 8, 9, Bax, p53, and the down-regulation of other antiapoptotic proteins, such as Bcl2 and poly (ADP-ribose) polymerase (PARP), honey is considered a good inducer of apoptosis. Another mechanism for honey in acting against cancer cells is the arrest of the cell cycle, by modulation of some molecules, such as cyclooxygenase and some kinases, or the induction of MOMP, promoted especially by flavonoids, which cause the release of intramembrane proteins into the cytosol, resulting in cell death. Indeed, the permeabilization of mitochondrial membrane is an early event that leads to the activation of the intrinsic mitochondrial pathway, which induces several processes, including the release of certain proteins such as cytochrome C (cytC), potentially cytotoxic, causing cell death [67]. 

The role of ROS and oxidative stress in cancer is still controversial, since it is unknown if it has a stimulatory or inhibitory effect. However, it would appear that the inhibition of tumor growth is still linked to the antioxidant properties of honey [68].

Finally, honey is able to counteract chronic inflammatory processes, which increase the risk of cancer. Two important factors of inflammatory pathway in cancer are nuclear factor kappa B (NF-kB) and mitogen-activated protein kinase (MAPK), which are involved in the up-regulation of some pro-inflammatory mediators such as interleukin 1 (IL-1), IL-6, and TNF-α [69], and some inflammatory proteins such as C-reactive protein (CRP), cyclooxygenase-2 (COX-2), and lipoxygenase-2 (LOX-2), playing an important role, not only in inflammation, but also in angiogenesis, a fundamental process of tumorigenesis [70]. 

Fauzi et al. investigated the effect of Tualang honey in breast (MCF-7 and MDA-MB-231) and cervical (HeLa) cancer cell lines, founding a cytotoxic effect, mainly due to a reduction of mitochondrial membrane potential, and to the activation of pro-apoptotic proteins such as caspase 3 and 9 [71]. Another study has also been carried out in vivo, investigating the effect of Manuka and Tualang honey on breast cancer. It was demonstrated a reduction of tumor growth, tumor grading, estrogenic activity, and hematological parameters. In addition, an increase in the expression of pro-apoptotic proteins, such as Caspase 9 and p53, and the involvement of some proteins of the inflammation pathway, such as TNF-α and COX-2 have been demonstrated [72].

Afrin et al. investigated the in vitro effect of Strawberry tree honey (STH) and Manuka honey against HCT-116, cells of human colon adenocarcinoma, and the LoVo metastatic cell line. They found an increase in intracellular ROS production, and an antiproliferative effect on both cell lines. A greater cytotoxic effect has been highlighted for STH, which presented a bigger amount of phytochemicals and antioxidant properties; these findings demonstrate that the cytotoxic effect of this honey could be related to the amount of polyphenols present in this matrix [73,74,75]. Also Jaganathan and Mandal studied the effect of some types of Indian honeys against colon cancer cell line (HCT-15 and HT-29), finding a cytotoxic effect of these honeys executed through the cell cycle arrest in subG1 phase and through the induction of apoptosis [76].

Another study also demonstrated an in vivo anticancer effect on colon cancer in Sprague Dawely rats injected with *N*-Nitroso-*N*-methylurea (NMU), acting primarily on inflammation and oxidative stress, contrasting the increase in nitric oxide (NO) and malondialdehyde (MDA), which had instead occurred in animals that had not received the honey-based supplement [77].

Tsiapara et al. studied the effect of three types of Greek honey (thyme, pine, and fir honey) on prostate cancer cells (PC-3) and endometrial cancer (Ishikawa), showing a reduction in vitality, and an increase of pro-apoptotic and apoptotic proteins [78]. Similar effects have been demonstrated on the same prostate cellular line with an Iranian honey. Further studies have been carried out to identify the compound responsible for this antiproliferative activity, and the chrysin, a flavonoid widely found in different types of honey, has been identified [79]. 

In liver cancer cells (HepG2), honey treatment led to the suppression of angiogenesis, induction of apoptosis and inhibition of cell proliferation [80]. A protective effect of honey has been proven also in vivo, on rats with diethylnitrosamine (DEN)-induced hepatic cancer, with up-regulation of p53 [81]. Similar effects have been found also for bladder cancer, both in vitro (T24, RT4, 253J) and in vivo on mice in which bladder cancer cells (MBT2) were implanted subcutaneously in the abdomens. The results showed an inhibitory effect on cellular proliferation, and on tumor growth with a reduction in the final tumor volume in mice treated with honey [82]. 

The antiproliferative and apoptotic effect of honey, and its polyphenols on human renal cancer cell lines (ACHN) was investigated, confirming the inhibitory activity of honey on these renal adenocarcinoma cells [83]. The effects of honey were also investigated for melanoma, both in vitro and in vivo. Pichichero et al. studied acacia honey’s activity on human melanoma (A375) and murine (B16-F1) cell lines. In both cases, an antiproliferative effect of honey has been demonstrated, mainly due to chrysin that mediated the cell cycle arrest in the G0/G1 phase [84]. Regarding in vivo study, Manuka honey, administered intravenously in mice where murine melanoma tumor cells (B16F1) were implanted, induced strong proapoptotic activity in a dose and time-dependent manner, decreasing the final tumor volume. Furthermore, mice in which Manuka honey was given together with chemotherapy drug (Taxol), had a higher life expectancy compared to those who received only the chemotherapeutic agent [85]. Ghashm et al. investigated the effects of Tualang honey on oral squamous cell carcinomas (OSCC) and human osteosarcoma (HOS), confirming its antiproliferative and proapoptotic effects on both cell lines [86]. Acacia honey has demonstrated anti-tumor activity in lung cancer cells (NCI-H460), inhibiting cell proliferation by stopping the cycle in the G0/G1 phase, stimulating cytokines and downregulating Bcl2 and p53, thus acting as a proapoptotic [87]. Morales, and Haza studied the effect of three different types of Spanish honeys, two monofloral (Heather and Rosemary) and one polyfloral in human leukemia cell line (HL-60). Monofloral honeys, particularly Heather honey, demonstrated a greater cytotoxic effect, mainly due to the induction of apoptosis through a ROS-independent pathway [88].

### 5.3. Antidiabetic Effect

There are several evidences that demonstrate a beneficial effect of honey on type 1 and type 2 diabetes mellitus. The measurement of fructosamine, glycosylated hemoglobin, and glucose is common and fundamental in the practice of glycemic control in patients with diabetes mellitus [89].

Many studies about the effect of honey against diabetes have been performed in vivo in rabbits and rats. In this context, a diet supplemented with honey was able to reduce glucose concentration in the serum of diabetic aloxantine-induced rats and streptozotocin (STZ)-induced rats [90]. Regarding fructosamine and glycosylated hemoglobin, the data are rather limited, although one study has showed that the combination of honey with some antidiabetic drugs, such as metformin and glibenclamide, leads to a reduction in glucose serum concentration but also in fructosamine [91].

Clinical studies have revealed that honey consumption, unlike other sweeteners, reduces the postprandial glycemic response in diabetic and non-diabetic volunteers, lowering the glucose serum concentration in patients with type 1 and type 2 diabetes [92]. 

Various evidences attribute the antidiabetic and the hypoglycemic capacity of honey to its antioxidant ability in relation to its dose; indeed, the pathogenesis of diabetes mellitus, especially type 2, seems to be closely associated with the presence of oxidative stress and ROS in various organs and tissues [93]. An increase of glucose absorption by adipose tissue and muscles raises ROS production, contributing to oxidative stress, mechanism that influences the synthesis of glycogen and glucose uptake. Additionally, oxidative stress can cause insulin resistance through the impairment of insulin signaling pathway, which can be restored by honey treatment [94]. Even in pancreatic β-cells, oxidative stress plays an important role, compromising their functionality, with a consequent incorrect insulin secretion and an increase in apoptosis of β-cells. Generally, it has been proven that the scavenger activity of honey improves pancreatic oxidative stress [95].

In diabetes mellitus, also lipid metabolism, is compromised, showing a high presence of low-density lipoproteins (LDLs), which are oxidized and glycated in oxLDLs, leading to endothelial damages. Even in this case, the antioxidant activity of honey helps to prevent the lipid oxidative metabolism in patients affects by type 2 diabetes mellitus [96].

### 5.4. Protective Effects of Honey

#### 5.4.1. Cardiovascular System

Several studies have demonstrated an association between a reduced risk of cardiovascular disorders and consumption of foods enriched with some compounds also present in honey, such as flavonoids and vitamin C. The cardioprotective effect of flavonoids has been widely demonstrated, and it is due to various mechanisms: (i) the reduction of the activity of blood platelets, (ii) the prevention of oxidation of LDLs, and (iii) the improvement of coronary vasodilatation [97].

The reduction of the activity of blood platelets has been demonstrated in vitro by Ahmed et al. who have investigated the effects of different types of honey on platelet aggregation and coagulation. Honey inhibited coagulation through all three coagulation cascades (intrinsic, extrinsic, and the common cascade) and decreased fibrinogen levels. For all these reasons, honey can be considered excellent for counteracting the process of atherosclerotic plaques formation that can lead to the development of cardiac disorders. In the pathogenesis of the atherosclerotic plaques, lipid peroxidation also plays a fundamental role [98]. It is confirmed that the honey phenolic compounds have a preventive and protective effect towards the damaging action of free radicals, counteracting, as explained, lipid peroxidation [99,100].

#### 5.4.2. Nervous System

Regarding the protective effect of honey in the nervous system, polyphenols play a central role. The scavenger activity against ROS, which are neurotoxic, and they can counteract various neurologic pathologies involved in aging. In addition, the deposition of misfolded proteins, such as beta amyloid, is the basis of some age-related neurological pathologies. Polyphenols are able to counteract this pathological accumulation [101].

Studies on the anti-hypnotic, anxiolytic, anticonvulsant, and antinociceptive of honey have been performed on mice and rats, and a real neuropharmacological effect has been demonstrated. In a study performed by Akanmu et al., the neuropharmacological effects of different types of Nigerian honey were studied in mice subjected to different behavioral stimuli. The anti-hypnotic effect of honey was investigated by the oral administration of honey and subsequently pentobarbital, a short-acting barbiturate. In animals that had received honey, the sleep time was reduced, anxiety was improved, and convulsions decreased [102]. The antinociceptive effects of different honey samples have been also demonstrated. Mice that had received an oral administration of honey showed an increase in their pain thresholds. This analgesic activity of honey is probably mediated by opioid receptors [103].

#### 5.4.3. Respiratory System

Even in popular medicine, honey is commonly utilized as a coughing sedative. Scientific studies regarding the protective activity of honey in respiratory system mainly concerns asthma. Asthma is a chronic inflammatory disease that is characterized by generally reversible obstruction of the lower airways, often as result of activity of allergens [104]. Kamaruzaman et al. have shown that honey inhalation is able to reduce inflammation of the lower airways in a rabbit model of ovalbumin-induced chronic asthma. Honey was capable of both preventing and improving the structural changes that occur following the induction of asthma through the allergen. It is known that honey is also able to reduce the number of inflammatory cells that are present in the fluid deriving from bronchoalveolar lavage, also inhibiting the bronchial hyperplasia of the goblet cells [105]. 

Finally, the role of honey in co-treatment with standard drugs in allergic rhinitis in subjects recruited from an otolaryngology clinic was investigated. The ingestion of honey in high doses (1 g/kg body weight daily for four weeks) improved the overall symptoms up to one month after the end of treatment [106].

#### 5.4.4. Gastrointestinal System

A protective effect on the gastrointestinal system has been also demonstrated. An important antimicrobial activity against *Helicobacter pylori*, that is responsible for gastroduodenal ulcers, has been demonstrated in vitro. The antimicrobial activity of different types of honey has been evaluated isolating *H. pylori* from patients with gastric diseases, and its susceptibility to the different types of honey was evaluated by measuring MIC and MBC. All the examined honeys possessed a high antibacterial activity with evident therapeutic potential [54]. 

Clinical studies have similarly shown an improvement in the treatment of infantile gastroenteritis, with a decrease in the duration of diarrhea, being also useful in the recovery of hydration post-gastroenteritis [107].

## 6. Physical Activity, Oxidative Stress, and Honey

A beneficial effect of honey has been shown in athletes, where if a moderate and regular exercise is able to counteract oxidative stress, an intense and prolonged physical activity can lead to an over-production of ROS that is often associated with a higher risk of incurring muscular injuries and also incurring a decrease in sporting performance [108]. The relationship between oxidative stress and sport is very complex, because, while dangerous, a release of free radicals is necessary to stimulate the up-regulation of endogenous antioxidant defenses. In recent years the consumption by athletes of supplements rich in antioxidant compounds has increased, but a natural contribution of these compounds through the diet is more recommended [109]. In this context, several studies have been carried out to explore the effect that honey consumption has in athletes; investigations have been performed both on murine models and on athletes practicing different sports.

Krisnanda investigated the effect of honey-based supplementation in male Wistar rats (Rattus norvegicus) subjected to carry out moderate-intensity physical activity (70% of maximum volume of oxygen VO2 max). After seven days, the value of one of the major markers of oxidative stress, MDA, was reduced in plasma by 35.52% in the group of rats that received honey (5 g/kg body weight once a day) [110]. On the other hand, Mosavat et al. studied the effect of eight weeks of honey supplementation (Tualang honey, 1 g/kg body weight per day) in female rats subjected to jumping exercise with different intensities on plasma levels of cortisol, which resulted in an increased in rats subjected to physical exercise compared to sedentary ones, with a lower level in the group subjected to 20 jumps a day compared to 80. Furthermore, in rats that received honey-based supplementation the increase of stress hormone levels was considerable lower [111].

About studies performed on athletes, the effects of Manuka honey on 32 healthy volunteers subjected to a short but intense exercise on cycle ergometer were explored. The determination of MDA levels in serum was performed 10 min before physical exercise and 30 min and 4 h after it. A significant decrease was noted in subjects who had consumed honey (1 g/kg body weight daily for 1, 2 or 3 weeks) before making physical exertion, with a greater difference for those volunteers who had used it for three weeks [112].

A very important study was carried out by Tartibian and Maleki, who examined the effects of honey in 39 road cyclists who participated to eight weeks of intensive cycling training. Some fundamental markers of oxidative stress (ROS, MDA) and antioxidant defenses (superoxide dismutase (SOD), catalase (CAT), total antioxidant capacity (TAC)) were examined in seminal fluid. The measurements were carried out at different times: at baseline and at 12, 24 and 24 h after the last training session, every four weeks. After four weeks and after eight weeks, ROS and MDA levels were significantly increased, while SOD, CAT, and TAC levels in the group supplemented with the artificial sweetener were decreased. In the group that received honey supplementation (70 g), the increase in markers of oxidative stress was much lower than in the placebo, and the levels of antioxidants were significantly higher [113]. Ahmad et al. examined the effect of high (1.5 g/kg body weight) and low doses (0.75 g/kg body weight) of Tualang honey in 20 female athletes that were involved in different competitive sports; the quantities of total phenolic content (TPC), MDA, and ROS were analyzed, and the antioxidant activity (ferric reducing antioxidant power (FRAP)) was monitored in the plasma of these athletes after 30 min, 1, 2 and 3 h after the administration of honey. The results showed that there was not significant difference between the two different doses and that the greatest antioxidant capacity was observed in both cases 2 h after the honey intake [114].

## 7. Infant Botulism and Other Toxic Compounds in Honey

Infant botulism is an acute condition that affects infants (0–1 year). The first report about this disease was in 1976 in USA. The microorganism *Clostridium botulinum*, an anaerobic Gram positive bacillus, is the main organism responsible for infant botulism, principally type A. In infant botulism, microbial spores are ingested [115], and they find the ideal conditions for the germination in the colon of children, where bacterial microflora are not yet well developed. Here, the spores germinate into the vegetative form, and in this phase they are able to produce toxins. From the intestinal tract, the botulinum neurotoxin reaches neuromuscular plaques via blood, where it works by preventing the release of acetylcholine, which determine the appearance of clinical symptomatology: descending flaccid paralysis that initially affects the musculature of the cranial nerves and can progress to respiratory arrest [116].

Honey is the most incriminated food as a vehicle for infant botulism. The contamination of honey can occur either in a beehive, or in the secondary stages of processing. Honey is considered to be one of the safest foods at a microbiological level, due to its high acidity for the presence of organic acids, and the low A_w_, which is necessary for the survival of microorganisms but not of spores, which manage to survive even in a hostile environment like honey [117].

Several studies have been carried out to verify the presence of *C. Botulinum* in honey: for example a Turkish work [118] analyzed 48 different types of commercial honey, and six of 48 (12.5%) resulted positive for the presence of *C. botulinum* spores. Gücükoğlu et al. have investigated 150 different types of honey and four of these were positive for the presence of neurotoxins of *C. botulinum* type A [119]; also in Brazil [120], the presence of spores was investigated in different samples of honey and three of 100 analyzed (3%) were positive; similar results were also obtained by Midura et al., which found nine honeys out of 90 (10%) [121] and Nevas et al., who analyzed 190 different types of honey and found that 20 of those were positive for the quiescent form of the microorganism (11%) [122]. 

There are also other toxic compounds that can be found in honey, which are not naturally present in it, but they derive from the industrial processes of heating and storage. 5-HMF is a compound that has been found in some honey samples, especially in those stored for a long time. In fact, high concentrations of 5-HMF are indicative of honey subjected to overheating or poor storage conditions, and may indicate that it is an old honey. Furthermore, the analysis of this compound is also used to monitor a possible adulteration of honey with inverted sugars [123]. This compound could be mutagenic, carcinogenic, and cytotoxic if taken in high doses [124]; for this reason the Codex Alimentarius Standard Commission has established a maximum limit for 5-HMF in honey at 40 mg/kg, with an exception for those of tropical origin (80 mg/kg) [125]. In other studies, it has been seen that honey can be contaminated with heavy metals such as mercury, cadmium, lead, and arsenic, which are toxic and carcinogenic [126]. 

Finally, there are plants that can contain in their nectar toxic substances that are harmful for human health, such as *Rhododendron ponticum* or *Azalea pontica* (honey derived from these plants is called “Mad honey”) that contain alkaloids, flowers from Andromeda may contain grayanotoxins that can cause paralysis of the arts [127]. Other toxic compounds found in honey that are derived from nectar may be hyoscyamine (from *Datura* sp. [128], hyoscine (from *Hyoscamus niger* [129], saponin (from *Serjania lethalis* [130], strychnine (from *Gelsemium sempervirens* [131], and others.

## 8. Conclusions

Honey is a natural product, and a source of amino acids, proteins, enzymes, essential minerals, vitamins, and bioactive compounds such as phenolic compounds, that possess interesting in vitro and in vivo biological properties. For example, it is effective in contrasting microbial infection, it has the potential to hinder malignant cellular proliferation through the modulation of several molecular pathways. At the same time, its consumption reduces the plasma level of fructosamine, glycosylated hemoglobin, and glucose in patients with diabetes mellitus, and it ameliorates several risk factors of cardiovascular disease; it exerts protective effects also in the nervous, respiratory, and gastrointestinal system. On the other hand, it is important to consider that it may contain some toxic compounds that should be avoided mainly in childhood. A deeper understanding of the factors and the mechanisms of honey effect will be of crucial importance to promote the consumption of this healthy food in the general population, to promote a healthy lifestyle and to prevent the most common pathology.

## Figures and Tables

**Table 1:** Chemical composition of the most commonly consumed types of honey. Adapted from Escuredo et al.

Component	Amount in 100 g of Honey
Water	16.9–18 g
Carbohydrates (total)	64.9–73.1 g
Fructose	35.6–41.8 g
Glucose	25.4–28.1 g
Maltose	1.8–2.7 g
Sucrose	0.23–1.21 g
Proteins, vitamins, amino acid and minerals	0.50–1 g

**Table 2:** Common phenolic acids and flavonoids in honeys.

Presence of Phenolic Compounds in Different Honeys		
Flavonoids		
Apigenin	C_15_H_10_O_5_	AH, TH, STH
Catechin	C_15_H_14_O_6_	TH, PH
Chrysin	C_15_H_10_O_4_	MH, AH, TH, HH, THH, RH
Galangin	C_15_H_10_O_5_	MH, AH, STH, HH
Genistein	C_15_H_10_O_5_	AH
Isorhamnetin	C_16_H_12_O_7_	MH
Kaempferol	C_15_H_10_O_6_	MH, AH, TH, STH, THH, RH
Luteolin	C_15_H_10_O_6_	MH, AH, TH, STH, THH, RH
Myricetin	C_15_H_10_O_8_	AH, HH, THH
Pinobanksin	C_15_H_12_O_5_	MH, AH, STH, RH
Pinocembrin	C_15_H_12_O_4_	MH, AH, STH, RH
Quercetin	C_15_H_10_O_7_	MH, AH, CH, THH
Rutin	C_27_H_30_O_16_	STH
Phenolic Acids		
2-*cis*,4-*trans* Abscisic acid	C_15_H_20_O_4_	STH
2-Hydroxycinnamic acid	C_9_H_8_O_3_	TH
Caffeic acid	C_9_H_8_O_4_	MH, AH, TH, THH
Chlorogenic acid	C_16_H_18_O_9_	AH, HH, THH
Cinnamic acid	C_9_H_8_O_2_	TH, STH, CH, HH, THH
Ellagic acid	C_14_H_6_O_8_	HH
Ferulic acid	C_10_H_10_O_4_	MH, AH, HH, THH
Gallic acid	C_7_H_6_O_5_	MH, AH, TH, HH, THH, PH
*p*-Coumaric acid	C_9_H_8_O_3_	MH, AH, TH, HH, THH, RH, PH
*p*-Hydroxybenzoic acid	C_7_H_6_O_3_	CH, HH
Protocatechuic acid	C_7_H_6_O_4_	HH, PH
Sinapic acid	C_11_H_12_O_5_	HH
Syringic acid	C_9_H_10_O_5_	MH, AH, TH, STH, HH, THH
Vanillic acid	C_8_H_8_O_4_	AH, HH

Manuka Honey (MH); Acacia Honey (AH); Tualang Honey (TH); Strawberry Tree Honey (STH); Clover Honey (CH); Heather Honey (HH); Thyme Honey (THH); Rosemary Honey (RH); Pine Honey.

**Table 3:** Some examples of antimicrobial activity of honey.

Bacterial Strain	Clinical Importance
*Helicobacter pylori*	Peptic ulcer, gastric malignancies, chronic gastritis
*Pseudomonas aeruginosa*	Diabetic foot ulcer, wound infection, urinary infections
*Escherichia coli*	Urinary tract infection, diarrhea, septicemia, wound infections
*Mycobacterium tuberculosis*	Tuberculosis
*Staphylococcus aureus*	Community acquired and nosocomial infection
*Proteus* spp.	Septicemia, urinary infections, wound infections
*Salmonella enterica*	Enteric fever
*Acinetobacter baumannii*	Infection through open wounds, catheters, and breathing tubes
*Vibrio cholerae*	Cholera
